# Supplementary material for: Psychosocial Distress in Parents with Children Awaiting Surgery during the COVID-19 Pandemic
Source: Children (Basel). 2022 Jan 9;9(1):87. doi: 10.3390/children9010087 (PMC8774209; doi:10.3390/children9010087)
Supplement: Supplementary file 1 [file children-09-00087-s001.zip › children-1450645-supplementary.pdf]

Supplemental Material

Supplemental Figure S1. Semi-structured Interview Guide.

1. Before we get started, I wonder if you can tell me what is happening with you during this time? Family life, work life, changes that have happened in your life due to COVID-19?

2. Tell me more about your understanding of your child's diagnosis (for example, ear infections, hearing loss, problems with eating, problems with moving).

3. What surgery was your child supposed to have that has been delayed (for example, ear tubes, adenoidectomy, cleft palate repair)?

4. Tell me about your experience since you heard your child's surgery was delayed because of the COVID-19 pandemic.
   a. How were you told?
   b. What was your initial reaction?

5. Did you have concerns at that time about your child's surgery being delayed?
   a. IF YES: Tell me more about your concerns.

How are you feeling now about your child's surgery being delayed?

6. What has been most difficult for you and your family during this time?

7. How would you describe your emotional well-being right now?

Worry, stress
Fear of progression of your child's diagnosis
Frustration, confusion, anger
Loneliness
Anxiety
Depression

8. Has the delay in your child's surgery led to changes in your physical health or behaviours?

Sleep disturbance
Irritability
Appetite changes
Headaches

9. Have you spoken to your surgeon or health care team about your concerns or your well-being?

a. IF YES: What did they say?
b. IF NO: Why not?

10. How are you managing during this time?
    Speaking with the ear, nose and throat (ENT) care team
    Speaking with your primary care provider
    Spouse/partner/people in your household
    Video chatting with family/friends
    Prayer
    Physical activity / exercise / yoga
    Mindfulness / meditation
    Eating healthy
    Maintaining routine
    Hobbies
    Any other ways

11. Thinking about when you get your child's surgery date – what do you think about going to the hospital around the time of COVID?

Concerned?

What are you concerned about?

12. Would you consider changes in child's treatment plan because of these delays, if your surgeon were to bring alternatives up? For example, undergoing ongoing monitoring to see if your child's diagnosis will improve over time rather than going to surgery right away?
    If that were possible, what would you think about it?

13. Is there anything else about your experience right now, specifically around your child's surgery being delayed, that we haven't discussed but you would like to tell me about?

Supplemental Figure S2. Reflexivity Statements

Patricia Leslie

As a research assistant, my primary role in this study was conducting and coding the interviews. My background is in Early Childhood Education and Infant and Early Childhood Mental Health. My gender is female. Training and guidance on qualitative research were provided by Dr. Urquhart. Personal goals for participating in the research were to gain experience in qualitative methodology and practice in the research environment, as well as learn more about how families cope when in distress and how we may be able to better support them. There was no relationship between me and any participant prior to study commencement.

David Forner

As the first author, my role in this study was that of study design, data analysis, and manuscript preparation. I am a white male in my 30's who was raised in Eastern Canada. My training background is in biology and chemistry (BSc Hon), medicine (MD), and clinical epidemiology (MSc). I am an otolaryngology – head & neck surgery resident physician and work with patients every day in a clinical capacity. Some of these patients have similar diagnoses to participants in the study. Furthermore, I have a strong interest in shared decision-making and related research, both clinically and in a research capacity. I have a strong desire to improve the lives of my current and future patients, and to identify areas in research which may assist in this goal. There were no relationships between myself and any of the participants before the study, and I have never acted as their physician.

Robin Urquhart

I am an experienced cancer health services researcher and hold the Canadian Cancer Society Endowed Chair in Population Cancer Research. My gender is female. I have >15 years of experience in qualitative and mixed methods research, and mentored Dr. David Forner and Patricia Leslie on this study. There was no relationship between me and any study participant.

Paul Hong

I am an experienced shared decision-making and outcomes researcher with more than 10 years of experience in mixed methods research. My gender is male. I mentored Dr. David Forner and Patricia Leslie during this study. I am an attending pediatric otolaryngology – head and neck surgery physician and work in a clinical capacity with patients and parents similar to those involved in this study. The children of some participants may have been under my care during the study.

Supplemental Table S3. Codes and Domains

Domain	Codes
**Communication & trust with/of health care team**	Communication with surgeon/health care team (pertaining to surgery)<br>● Informed of COVID delay (or not)<br>● Reassurance from team<br>Multiple surgical dates/delays<br>Communication with primary care provider<br>Trust in surgeon/heatlh care team
**COVID challenges**	Social isolation<br>Navigating the system<br>Inability to continue with normal routine<br>Isolation<br>Travel<br>Changes in employment<br>Changes in childcare/schooling
**Parent factors**	Parental understanding (of their diagnosis, need for surgery, etc)<br>Familiarity<br>Parental expectations<br>Wanting to "get it done"
**Parent health & well-being**	Emotional distress<br>Level of COVID-related worry<br>Fear of consequences due to the delay<br>Feeling delay has caused a delay in development<br>Fear of progression<br>Physical health and well-being<br>Lack of control<br>Minimizing their situation<br>The Unknown
**Coping mechanisms & supports**	Family / friend support<br>Keeping busy<br>Other coping mechanisms/supports<br>Spirituality - faith<br>Being physically active - exercise
**Hospital factors during COVID**	Hospital restrictions (e.g., no visitors)<br>Risk of catching COVID
**Other**	Alternatives to surgery
